# Resting parasympathetic activity is associated with malodor‐induced change in perceived foreignness of speakers

**DOI:** 10.1002/brb3.3249

**Published:** 2023-09-21

**Authors:** Kelly E. Faig, Elizabeth A. Necka, Karen E. Smith, Stephanie J. Dimitroff, Greg J. Norman

**Affiliations:** ^1^ Department of Psychology Hamilton College Clinton New York USA; ^2^ Department of Psychology The University of Chicago Chicago Illinois USA; ^3^ Department of Psychology Rutgers University‐Newark Newark New Jersey USA; ^4^ Department of Psychology University of Montana Missoula Montana USA

**Keywords:** behavioral immune system, parasympathetic nervous system, social perception

## Abstract

**Introduction:**

To protect against infection, individuals have evolved context‐dependent pathogen‐avoidant strategies, including selective social behaviors aimed at avoiding foreign individuals who may possess greater risk of infection. Parasympathetic nervous system (PNS) activity is associated with social engagement and regulation of the classical immune system but has not been widely investigated in relation to changes in intergroup perception and the behavioral immune system.

**Method:**

The current research investigated the relationship between parasympathetic activity and perceived foreignness of in and outgroup speakers during exposure to a pathogen‐relevant odor (butyric acid). High‐frequency heart rate variability was measured at rest and while participants rated foreignness of speakers with and without the odor present.

**Results:**

Findings show that exposure to the odor was associated with higher foreignness perceptions of outgroup speakers and lower foreignness perceptions of ingroup speakers. This effect was especially evident among individuals with higher resting parasympathetic activity.

**Conclusion:**

These results suggest that the PNS may play a role in changes in social perceptions during a behavioral immune response.

## Graphical abstract

Our research examined the involvement of the parasympathetic nervous system in one type of behavioral immune response: shifts in intergroup perceptions during presence of a pathogen threat. Results show that exposure to a pathogen‐relevant odor resulted in higher foreignness perceptions of outgroup speakers, especially among individuals with higher resting parasympathetic activity.

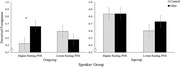

## 1 INTRODUCTION

To survive, organisms must be able to successfully recognize and respond to salient threats in the environment. One such threat is that of infection. Infectious illnesses account for almost one quarter of global deaths (WHO, 2020a). In the last 3 years, 6.9 million individuals have died from COVID‐19 (WHO, 2020b). Given the substantial costs associated with infection (Lochmiller & Deerenberg, 2000; Miles et al., 2020), humans and other organisms have evolved responses to reduce the risk of exposure to potential pathogens (Ackerman et al., 2018; Murray & Shaller, 2016). These adaptations, which include selective social behaviors aimed at avoiding individuals who may pose an increased risk of infection, are part of what is referred to as the behavioral immune system (BIS). The BIS collectively describes a set of physiological, psychological, and behavioral processes that protect against infection by promoting avoidance of pathogens (Schaller & Park, 2011). It also interacts with the classical immune system, or the physiological mechanisms that activate after infection (Ackerman et al., 2018; Gassen et al., 2018; Murray et al., 2019). Here, we integrate across functionalist theories of social behavior and autonomic regulation to investigate the role of the parasympathetic nervous system (PNS), a marker of flexible and adaptive motivational responding, and a key moderator of classical immunity (Pavlov et al., 2018; Porges et al., 2021), in one type of behavioral immune response: shifts in intergroup perceptions during a pathogen threat.

### 1.1 Social implications of the behavioral immune system

The BIS is thought to promote the avoidance of infected conspecifics through an increase in discriminating social perceptions, such as intergroup categorization processes (Oaten et al., 2011). Throughout evolutionary history, social contacts who lived in close proximity were likely to be exposed to similar pathogens and share comparable immune profiles, whereas those from geographically distant regions conferred a higher risk of having a novel contagious illness (Freeland, 1979; Markel, 1999; Schaller, 2011). Therefore, preferential interaction with kin and other ingroup members over foreign individuals and outgroup members may act as a protective mechanism against infection (Fincher & Thornhill, 2012). The idea that the BIS promotes certain types of social interactions over others is in line with functionalist perspectives on social cognition. These perspectives emphasize the role of flexible responses to threat that are context‐dependent (Miller et al., 2010; Neuberg & Schaller, 2016). Selective social behaviors that discriminate between in and outgroup members during an immune‐related threat may be one type of context‐dependent response that has facilitated successful avoidance of pathogens among ancestral populations.

Extant experimental research has found associations between intergroup biases and the BIS. For example, individuals who viewed pictures containing infection cues subsequently reported more negative attitudes toward immigrant populations that were rated as foreign (Faulkner et al., 2004). Persons exposed to a pathogen prime were more inclined to categorize individuals in neutral photographs as members of an outgroup (Makhanova et al., 2014). Additionally, self‐reported perceived vulnerability to disease has been correlated with ethnocentric attitudes (Navarrete & Fessler, 2006). More recently, priming the saliency of the COVID‐19 pandemic has been shown to increase support for travel bans in countries with high pandemic risk (Moran et al., 2021). However, other evidence suggests that the BIS does not uniformly promote avoidance of outgroup members (Aarøe et al., 2016; Bressan, 2021; Petersen, 2017; van Leeuwen & Petersen, 2018). For example, van Leeuwen and Petersen (2018) demonstrated that although the presence of a pathogen cue impacted reported comfort with contacting another person, group membership did not. These inconsistencies suggest that there may be more nuanced mechanisms involved in the effects of pathogens on intergroup behaviors.

One possibility is that the intergroup effects of the BIS depend on individual variability in perceptions of group membership. In a global society, there are vast differences in the social groups each individual is exposed to, and in the nature of those exposures. As such, subjective definitions of group membership may be more likely to influence behavior than indicators like race or ethnicity. For example, a recent reanalysis of van Leeuwen and Petersen's (2018) data demonstrated that when group membership was defined by participants’ perceived similarity to locals rather than by ethnicity, participants reported more discomfort contacting outgroup members, especially when a pathogen cue was present (Bressan, 2021). Participants’ perceived similarity to locals was also correlated with perceived health ratings of the individuals, indicating a link between perceptions of similarity and evaluations of infection risk. This suggests that the use of perception to define groups may be a more targeted approach than the use of other indicators. In further support of the role of perception, evidence shows that individuals who report greater sensitivity to pathogen cues—for example, increased disgust—tend to rate ingroup speakers as more similar to themselves, and outgroup speakers as more dissimilar, when exposed to a pathogen prime (Reid et al., 2012). This suggests that individual perceptual differences impact the extent that a situational pathogen prime activates the BIS and alters evaluation of group members, as defined by perceived linguistic distance to the self (Fincher & Thornhill, 2008; Reid et al., 2012). Yet, the detection of and ability to adaptively respond to salient immune threats may vary between individuals, and the identification of mechanisms underlying this variability is crucial to a comprehensive understanding of the BIS.

### 1.2 Parasympathetic immune regulation

The PNS represents one mechanism that may contribute to interindividual variation in the ability to detect and respond to potential social immune threats. As asserted by the polyvagal theory (Porges & Carter, 2017), and the neurovisceral integration model (Smith et al., 2017), the PNS is involved in social engagement and emotional and cognitive processes. The PNS consists of extensive projections to nearly all organ systems that facilitate rapid shifts in visceral activity allowing organisms to adapt in dynamic environments via coordination through prefrontal‐subcortical circuits critical to motivational responding (Åhs et al., 2009; Lane et al., 2009; Porges & Carter, 2017; Smith et al., 2017). PNS cardiac activity at rest has been shown to covary with activity in these central circuits (Thayer & Lane, 2009) and has been linked to variability in sensitivity to threat and safety cues in the environment. For example, individuals with lower resting PNS have shown impaired recognition of safety cues and hypervigilance to emotional stimuli (Brosschot et al., 2017; Park & Thayer, 2014). PNS activity is also thought to have specific implications for social behavior relevant to intergroup processes (Porges & Carter, 2017). Individuals with higher resting PNS have demonstrated greater ingroup affiliation in a minimal‐groups paradigm (Sahdra et al., 2015) and more accurate performance on a target detection task in the presence of outgroup‐stimuli distractors (Park et al., 2022). Together, this research suggests that PNS activity at rest can serve as a marker for capacity to generate flexible responses in dynamic intergroup contexts that motivate affiliative or attentional regulatory strategies.

In addition to the PNS's involvement in complex social regulatory processes, it plays a critical role in coordinating classical neuroimmune processes. The vagus nerve is the primary signaling component of the PNS. It is one of the principal means through which the immune system sends pathogen‐related information to the brain (Dantzer et al., 2008), and descending vagal signaling has been found to regulate systemic inflammatory processes (Pavlov & Tracey, 2012). This overlap in functionality, including involvement in social engagement and classical immune function, makes the PNS a likely effector supporting behavioral immune processes like discriminating social perceptions. Preliminary evidence is suggestive of PNS involvement in behavioral immune responses. In particular, increased PNS activity is associated with emotional responses linked to potential immune threats, like disgust (Ritz et al., 2005; Rohrmann & Hopp, 2008). Yet, explicit connections between PNS activity and the social behavioral immune responses have yet to be established. The present study aims to bridge this gap by explicitly assessing whether PNS cardiac activity moderates shifts in intergroup perceptions in the context of a salient immune threat.

### 1.3 The present study

In the current study, we investigated the role of the PNS in shifts in intergroup perceptions mediated by the BIS. Specifically, we assessed whether the presence of a pathogen‐relevant odor affected the perceived foreignness of in and outgroup speakers, and if resting and task‐related PNS activity was related to this perceptual difference. We hypothesized that during exposure to the odor, participants would rate outgroup speakers as more foreign, and ingroup speakers as less foreign. Given higher resting PNS activity is thought to be a marker of flexible and contextually adaptive motivational responding (Balzarotti et al., 2017), we expected that this effect would be especially evident among individuals showing higher PNS activity.

## 2 METHODS

### 2.1 Participants

Sixty‐two students from the University of Chicago participated in this experiment and were randomly assigned to odor or control conditions. The sample size is consistent with recommendations from power simulation studies for hierarchical linear models (HLMs) (Kerkhoff & Nussbeck, 2019). Post hoc power analyses (reported in Table S1) found that the primary effect was powered at 80.1%. Due to incomplete data and a preexisting health condition, three participants were excluded from all analyses, and one had behavioral data only. This participant was excluded from analyses using PNS measures but included for analyses focused only on behavioral data. Excluding this participant from behavioral analyses did not change results in a meaningful way. The remaining 59 participants (29 control/30 odor) were all fluent in English, and 48 participants reported English as their native language (24 in each condition). No participants reported any olfactory impairments. Forty‐three of these participants were female (20 control/23 odor), 5 were African American (1 control/4 odor), 19 Asian/Pacific Islander (9 control/10 odor), 31 White (15 control/16 odor), 1 Hispanic (1 control/0 odor), and 3 Biracial (3 control/0 odor); *M*_age_: 21.22 years, SD: 1.86. This study was approved by the University of Chicago's Institutional Review Board. All participants provided written consent prior to participation and were fully debriefed at the end of the experiment.

### 2.2 Procedure

**FIGURE 1:**
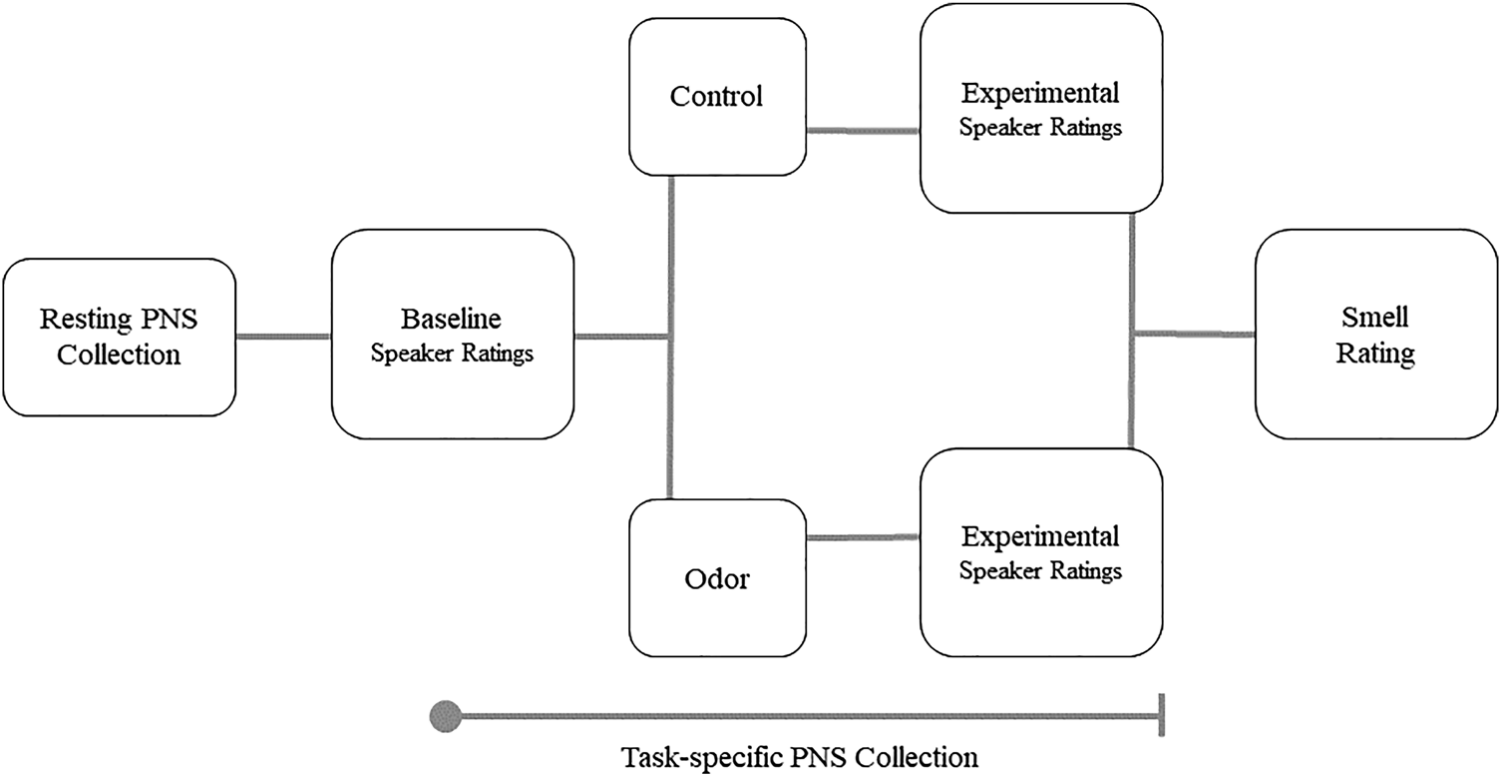
Diagram of study procedure. *Note*: All participants had resting parasympathetic nervous system (PNS) activity collected for a 5‐min period followed by baseline ratings of speaker foreignness. After baseline speaker ratings, participants were randomly assigned to the control condition or the odor condition. All participants completed a second set of speaker ratings in the experimental conditions (control/odor) followed by a smell rating of the room, which served as a manipulation check.

An overview of the experiment is shown in Figure 1. Participants were recruited via flyers and the SONA participant system at the University of Chicago. They were told that the study would consist of evaluating different visual, auditory, and olfactory stimuli. Upon arrival at the laboratory, participants were instructed to continue completing experimental tasks whether or not they detected any change in the smell of the room. First, they were asked to sit quietly for 5 min during a measure of resting PNS activity. Participants were then asked to rate accented speakers on perceived foreignness. After the baseline trial ratings, participants were exposed to an odor manipulation designed to activate the BIS due to its pathogen‐relevant and disgusting nature. This involved the experimenter opening vials containing the odor stimulus on a desk that was not visible to participants. Participants then rated a second set of accented speakers, while exposed to the odor (or no‐odor control). Following completion of each set of ratings (baseline and post‐odor manipulation), all participants rated how positive and negative they perceived the room to smell via an evaluative space grid (Cacioppo & Berntson, 1994; Norman et al., 2011) as a manipulation check. Participants also completed a demographics survey and individual difference measures (see Table S2).

#### 2.2.1 Ratings of accented speakers

Participants listened to 36 speakers (International Dialects of English Archive)^i^ with varying accents read a standardized script. Immediately after listening to each speaker audio clip, participants were asked to rate “How foreign does this speaker sound?” on a Likert‐type scale (1 = not at all, 9 = extremely), consistent with previous work (Reid et al., 2012). Audio clips were divided into two matched sets (see Supporting Information section for additional details). One set was administered at baseline and the other during the odor manipulation. To minimize habituation to the script, audio clips varied slightly in duration and in the specific portion of the script that was spoken. Audio clips were cut into 3‐, 6‐, or 9‐s segments (duration was counterbalanced across sets). Supplementary analyses show that duration did not impact reported results. Audio clips of speakers within each set were presented in a random order for each participant, and set order was counterbalanced between participants.

#### 2.2.2 Odor manipulation

Participants were randomly assigned to experimental (odor) or control (no‐odor) conditions. Conditions were identical except that, in the odor condition, participants completed the post‐odor manipulation ratings while exposed to butyric acid in the room. Butyric acid is a pathogen‐relevant odor cue. It is commonly produced by bacteria contained in vomit and rancid foods, and it elicits a putrid smell and reliably induces avoidance behavior (Jacob et al., 2003; Workman et al., 2009). For the odor condition, 250 μL of butyric acid at 50% dilution with filtered water was pipetted into four 2 mL vials. For both control and odor conditions, four identical vials were opened all at once at the start of the post‐odor manipulation trial on an adjacent desk not visible to participants.

#### 2.2.3 Parasympathetic nervous system activity

High‐frequency heart rate variability (HF‐HRV) was used to measure PNS activity. HF‐HRV measures the fluctuation in heart period associated with respiration. HF‐HRV has been demonstrated to be an index of parasympathetic cardiac control (Berntson et al., 1997). We chose to focus on HF‐HRV because lower frequencies reflect both parasympathetic and sympathetic activity (Berntson et al., 1997). Mindware HRV Analysis software (Mindware Technologies LTD; version 3.1.3) was used to derive HF‐HRV by spectral analysis of the interbeat interval series from the ECG. The interbeat interval series was time sampled at 4 Hz (with interpolation) to yield an equal interval time series. This time series was detrended (second‐order polynomial), end tapered, and submitted to a fast Fourier transformation. The spectral power was then integrated over the respiratory frequency band (0.12–0.40 Hz). HF‐HRV is represented as the natural log of the heart period variance in the respiratory band (in ms^2^) (Berntson et al., 1997). Data were preprocessed in accordance with published guidelines (Berntson et al., 1997) in 1‐min segments. For resting state HF‐HRV, 1‐min segments were averaged across a 5‐min resting period. For HF‐HRV during the ratings, 1‐min segments were averaged across each rating period (baseline and post‐odor manipulation). Audio clip specific activity was not examined because frequency‐based measures of PNS activity cannot be calculated for intervals shorter than 30 s (Berntson et al., 1997).

### 2.3 Data analysis plan

#### 2.3.1 Determining in and outgroup speaker groups

To account for individual differences in language experience and accented‐speech exposure, in and outgroup speaker groups were uniquely identified for each participant. Using the first set of foreignness ratings, we computed a mean baseline foreignness score for all participants. These baseline foreignness scores were used to divide odor manipulation speakers into in and outgroup speaker groups. Post‐odor manipulation, the speakers that participants evaluated as more foreign than their own baseline foreignness score (i.e., greater than within‐subject baseline foreignness) were categorized as the outgroup, and those that participants evaluated as less foreign than their baseline (i.e., less than within‐subject baseline foreignness) were categorized as the ingroup.

*Ingroup* = odor manipulation speakers rated below individual baseline mean foreignness

*Outgroup* = odor manipulation speakers rated above individual baseline mean foreignness

This allowed for the examination of the divergent effects of the odor manipulation on perception of in and outgroup speakers in a manner tailored to the unique perceived foreignness ratings of each participant. Participant categorization of speakers indicated that on average, they identified 7.5 speakers as ingroup members and 10.5 speakers as outgroup members across trials and conditions. For a table of the mean number of in and outgroup speakers identified by participants in each condition during baseline and post‐odor manipulation trials specifically, see Table S3. As a manipulation check of baseline speaker groups, in and outgroup speakers categorized during baseline trials demonstrated significant differences in perceived foreignness (*t*_89_ = −33.13, *p* < .001).

#### 2.3.2 Statistical analyses

Differences in baseline and post‐odor manipulation foreignness ratings were analyzed using HLMs fit by maximum likelihood estimation (Raudenbush & Bryk, 2002). Condition (odor/control), speaker group (ingroup/outgroup), and their interaction were included in the models as fixed factors. Random intercepts were modeled for participant and audio clip (Baayen et al., 2008). To examine foreignness ratings relative to each individual's baseline foreignness, the post‐odor manipulation ratings were subtracted from baseline. To test whether resting PNS activity statistically moderated any difference in the speaker ratings, resting HF‐HRV was added to the HLMs as an additional subject‐level fixed factor. HF‐HRV reactivity was examined as a change score from rest (HF‐HRV^TASK^—HF‐HRV^REST^). All analyses were also run controlling for age, race/ethnicity, and gender covariates. Continuous variables were standardized prior to analyses (i.e., *M* = 0, *SD* = 1) permitting use of regression coefficients for effect size estimates (Lorah, 2018). HLM models were run in R (R Core Team, 2023) using the lme4 package (Bates et al., 2015) and the car package (Fox & Weisberg, 2019). Interactions were examined by calculating simple slopes using the emmeans package (Lenth, 2023).

## 3 RESULTS

### 3.1 Manipulation checks

A repeated measures ANOVA was conducted (afex package, Singmann et al., 2023) to compare differences in the smell rating from baseline to post‐odor manipulation between participants in odor and control conditions. As expected, the ANOVA yielded a significant interaction effect between condition (odor/control) and trial (baseline/odor), (*F*_(1,57)_ = 42.46, *p* < .001). Participants in the odor condition rated the smell of the room as significantly more negative after the post‐odor manipulation trials when exposed to butyric acid, indicative of a successful odor manipulation. We found no significant differences in perceived foreignness between the two counterbalanced speaker sets in this sample (*t*_34_ = .04, *p* = .966), confirming the ratings from pilot testing (see Supporting Information section for additional details). All reported results remain unchanged when controlling for counterbalanced speaker sets.

### 3.2 Foreignness

**FIGURE 2:**
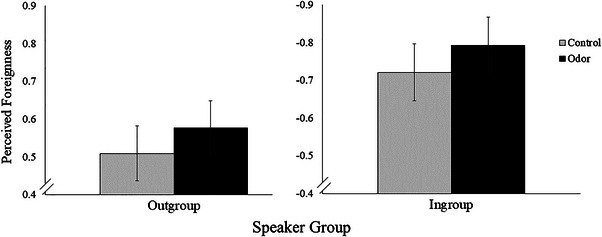
Exposure to a pathogen‐relevant odor predicts perceived foreignness of in and outgroup speakers. *Note*. Participants in the odor condition showed higher foreignness ratings of outgroup speakers and lower foreignness ratings of ingroup speakers compared to control participants (*β* = .14, *p* = .005, 95% CI [.04, .24]). Graph shows fitted values based on model predictions. Error bars reflect standard error of the mean.

First, we examined whether odor condition (odor/control) and speaker group (in/outgroup) influenced post‐odor ratings of perceived foreignness. As expected, there was a significant interaction between condition and speaker group (*β* = .14, *p* = .005, 95% CI [.04, .24]). Participants in the odor condition rated outgroup speakers as higher in perceived foreignness ratings and ingroup speakers as lower in perceived foreignness ratings (*β* = −1.37, *p* < .001) compared to control participants (*β* = −1.23, *p* < .001) (Figure 2). As expected based on the method by which we designated in and outgroup speakers, there was also a main effect of speaker group (*β* = 1.23, *p* < .001, 95% CI [1.15, 1.31]) confirming that outgroup speakers were rated as more foreign than ingroup speakers. The main effect of condition on perceived foreignness did not reach significance (*β* = −.07, *p* = .234, 95% CI [−.19, .05]).

### 3.3 HF‐HRV

**FIGURE 3:**
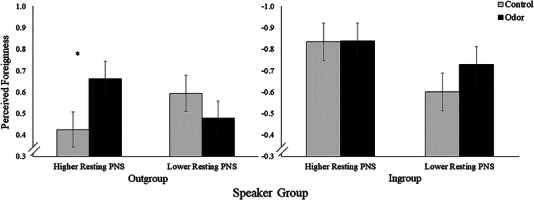
Resting parasympathetic nervous system (PNS) activity moderates the effect of odor on perceived foreignness of in and outgroup speakers. *Note*: Participants in the odor condition with higher resting PNS activity rated outgroup speakers as more foreign than participants with lower resting PNS in the same condition (*β* = −.18, *p* = .013). PNS was measured with high‐frequency heart rate variability. Higher and lower resting PNS are represented by one standard deviation from the grand mean for visualization purposes (continuous measure used in analyses). Graph shows fitted values based on model predictions. Error bars reflect standard error of the mean.

To examine whether resting PNS activity influenced the effects of odor on perceived foreignness ratings, resting HF‐HRV was added to the above model as a fixed factor (in addition to condition and speaker group). As hypothesized, there was a significant interaction among resting HF‐HRV, condition, and speaker group (*β* = .11, *p* = .027, 95% CI [.01, .21]). Analysis of simple slopes revealed that within the odor condition, participants with higher resting HF‐HRV had higher perceived foreignness ratings of outgroup speakers compared to participants with lower HF‐HRV (*β* = −.18, *p* = .013). However, there was no significant difference between odor condition participants with higher compared to lower HF‐HRV on odor manipulation ratings of ingroup speakers (*β* = .11, *p* = .162) (Figure 3). A main effect of resting HF‐HRV was also revealed, such that participants with lower resting HF‐HRV rated speakers as more foreign post‐odor manipulation, regardless of group status and odor condition (*β* = −.12, *p* = .013, 95% CI [−.21, −.03]). Replicating the original analysis, the main effect of speaker group again showed that participants rated outgroup speakers as more foreign than ingroup speakers (*β* = 1.23, *p* < .001, 95% CI [1.14, 1.31]), serving as a manipulation check confirming appropriate designation of in and outgroup speakers. Additionally, the two‐way interaction between condition and speaker group was again significant, indicating that the odor group showed higher foreignness ratings of outgroup speakers and lower foreignness ratings of ingroup speakers compared to the control group (*β* = .13, *p* = .01, 95% CI [.03, .23]). The main effect of condition (*β* = −.07, *p* = .256, 95% CI [−.18, .05]) and all other two‐way interaction effects failed to reach significance (*p*s > .05). In models using HF‐HRV reactivity instead of resting HF‐HRV, HF‐HRV reactivity did not interact with condition and speaker group nor was there a significant main effect or any significant two‐way interactions (*p*s > .05). All reported results remain unchanged when controlling for age, race/ethnicity, and gender covariates in the models.

## 4 DISCUSSION

The current study adds to a growing body of literature demonstrating that activation of the BIS via pathogen primes is associated with shifts in intergroup perceptions. Our finding that individuals exposed to the pathogen‐relevant odor rated outgroup speakers as more foreign is in line with previous research suggesting that pathogen threats are linked to more frequent categorization of neutral individuals as outgroup members (Makhanova et al., 2014) and perception of outgroup speakers as less similar to the self, with ingroup speakers perceived as more similar (Reid et al., 2012). Our study additionally extends this research, finding that PNS regulation may contribute to variability in the extent to which a pathogen threat shifts intergroup perceptions, such that increases in rated foreignness of outgroup speakers in the odor condition were most pronounced for individuals with higher levels of resting PNS cardiac activity.

Our behavioral findings suggest that exposure to a pathogen‐relevant odor, compared to control, led to small increases in perceived foreignness ratings of outgroup speakers and small decreases in perceived foreignness ratings of ingroup speakers. Functionalist theories of social cognition emphasize the importance of flexible and context‐dependent responses to social threats (Miller et al., 2010; Neuberg & Schaller, 2016). Historically, outgroup members were more likely to possess unfamiliar, potentially deadly pathogens (Freeland, 1979; Markel, 1999; Schaller, 2011). Therefore, responses promoting preferential contact with ingroup members compared to outgroup members during a pathogen threat may have evolved as a form of self‐protection (Fincher & Thornhill, 2012; Schaller, 2011). Existing work has shown that infection‐related primes are associated with behavioral changes linked to outgroup avoidance and ingroup approach (Makhanova et al., 2014; Miller & Maner, 2012; Reid et al., 2012). Our research expands on these findings, suggesting that olfactory pathogen‐relevant stimuli may result in behavioral shifts via subtle changes in how individuals perceive cues associated with outgroup members (e.g., foreign speech). Changes in perceived foreignness in particular may be representative of an increase in the perceived linguistic distance between participants and outgroup members (Reid et al., 2012). Increased linguistic distance could promote avoidance of those that may be associated with a higher risk of infection. Similarly, a concomitant decrease in the perceived linguistic distance between participants and ingroup members may facilitate affiliative and approach‐related behaviors aimed at garnering sources of support if actual infections were to occur (Imada & Mifune, 2021). This is consistent with research that found that injection with an immune stimulant (lipopolysaccharide) elicited increased desire to spend time with support figures (Inagaki et al., 2015). The degree of ingroup‐related approach during a pathogen threat may also vary by culture, as ingroup derogation has also been demonstrated (Wu et al., 2019). Future work may further investigate mechanisms other than perceived linguistic distance that may underlie discriminatory social perceptions promoting affiliation with ingroup members and avoidance of outgroup members during immune‐related threats.

Our findings show that shifts in perceived foreignness were most pronounced for individuals with higher resting PNS activity. This aligns with theoretical proposals that resting PNS cardiac activity reflects individual ability to flexibly respond to dynamic environmental events in a context‐dependent manner (Porges & Carter, 2017; Smith et al., 2017). Our results suggest that, in the context of a potential immune threat, higher resting PNS activity is associated with increased sensitivity to social cues that may be indicative of heightened risk of infection. Our data add to evidence implicating the PNS in changes related to the BIS, including emotional reactions to immune‐relevant stimuli (Ritz et al., 2005; Rohrmann & Hopp, 2008). Recent work has suggested a synergistic or coactive relationship between the classical immune system and the BIS. This relationship is characterized by concomitant increases in both systemic immune signaling and behavioral responses that may minimize exposure to pathogens (Azlan et al., 2017; Fessler et al., 2005; Oaten et al., 2016). The PNS is highly involved in this coactivity, as evidenced by vagally‐mediated sickness behaviors like decreased food intake, decreased social interactions, and increased sleep when the body is fighting infection (McCusker & Kelley, 2013). Our data suggest that the PNS may also influence coactivity between classical and behavioral immune responses in the context of preemptive pathogen threat, prior to infection. Additional work has described a reciprocal or compensatory relationship between the classical immune system and the BIS in which downregulated immune function is associated with elevated motivation to avoid pathogens (Bradshaw et al., 2022; Cepon‐Robins et al., 2021; Gassen et al., 2018). Future work will be necessary to elucidate the complexities behind reciprocal and coactive response patterns of classical and behavioral immune systems (for more discussion see Ackerman et al., 2018), as well as the role of the vagus nerve in these processes.

Although not the primary question of the current research, we also found that individuals with lower resting PNS activity demonstrated higher perceived foreignness ratings regardless of speaker group and condition. This may relate to recent work suggesting that lower resting PNS activity is associated with increased feelings of unsafety and fear, especially in social contexts (Brosschot, 2017; Koenig, 2020; Smith et al., 2017). Heightened perceptions of foreignness among individuals with lower resting PNS could highlight a greater sensitivity to interpersonal differences that may ultimately affect social engagement. Indeed, prior work has reported that lower resting PNS also predicts increased social stress (Brosschot et al., 2018) and less accurate performance on an attention task involving outgroup‐stimuli distractors (Park et al., 2022). On the other hand, higher resting PNS is associated with increased cooperation (Beffara et al., 2016) and increased ingroup affiliation (Sahdra et al., 2015). Given these connections between resting PNS activity and social tendencies, replication of the association between lower resting HF‐HRV and increased perceived foreignness, and examination of relations to downstream social outcomes are promising avenues for future research.

## 5 LIMITATIONS AND FUTURE DIRECTIONS

The current research can be expanded on in future studies in several ways. First, the population that was studied was primarily comprised of fluent English‐speaking undergraduates and individuals identifying as female. Replication in a larger sample consisting of a more diverse and representative population is critical to determine whether the findings are generalizable. Additionally, because of large potential differences in participant backgrounds and idiosyncratic factors influencing speaker perceptions between individuals (Drager, 2010), we used baseline ratings to determine individuals’ own in/outgroup designations (Bressan, 2021). Although this allowed us to better capture individual variability in perceptions of group status, it also meant that we were not able to examine change in odor ratings, making it possible that differences in perceived foreignness between the groups are due to other confounding factors. However the lack of any differences in baseline foreignness ratings suggests that this is not the case. Last, as the present study employed an odor manipulation, future work will be necessary to determine whether our results are specific to olfactory‐induced shifts or are part of a more generalized multisensory system for recognizing and responding to infection in conspecifics (Regenbogen et al., 2017).

## 6 CONCLUSIONS

Overall, this work provides valuable insight into the complex relationship among the BIS, resting PNS activity, and social perception. In particular, these findings suggest that the PNS may play a role in social perceptions associated with the BIS by facilitating flexible, context‐dependent responses within dynamic environments (Balzarotti et al., 2017; Porges, 2003). The ability to track differences in perceived foreignness across contexts may permit successful mobilization of infection‐relevant avoidance behaviors. Future research should examine how these shifts in intergroup perception translate to changes in motivated behaviors. Results also point to a need for future work to investigate the intricacies of the relationship between the PNS and both classical and behavioral immune responses. Current work is beginning to uncover connections between the PNS and integrative measures health that reflect both behavioral and classical immunity, like self‐rated health (Jarczok et al., 2015). Examination of how PNS regulation interacts with other aspects of health to influence ingroup approach and outgroup avoidance may clarify the role the BIS plays in social behavior. Inclusion of the PNS may further elucidate how rapid communication and inhibitory control between the brain and target projection organ systems in the body (including the heart and immune system) facilitate positive health outcomes and effective self‐regulation. This type of research could provide insights into variability in responses to pathogens and other self‐protective behaviors in response to threats to the immune system.

## AUTHOR CONTRIBUTIONS

**Kelly E. Faig**: Conceptualization; data curation; formal analysis; investigation; methodology; project administration; validation; visualization; writing—original draft, writing—review and editing. **Elizabeth A. Necka, Karen E. Smith, Stephanie J. Dimitroff**: Data curation; formal analysis; validation; visualization; writing—review and editing. **Greg J. Norman**: Conceptualization; data curation; formal analysis; funding acquisition; investigation; methodology; resources; software; supervision; validation; visualization; writing—original draft; writing—review and editing.

## CONFLICT OF INTEREST STATEMENT

The authors have no conflicts of interest to disclose.

## FUNDING INFORMATION

National Institute of Mental Health [T32MH018931‐30 (KES)]

### PEER REVIEW

The peer review history for this article is available at https://publons.com/publon/10.1002/brb3.3249.

## Supporting information

Supporting Information

## Data Availability

All associated data and code is available on the Open Science Framework (OSF; https://osf.io/j83xw/).

## NOTE

i The recordings used in this project are used by special permission of the International Dialects of English Archive, online at http://www.dialectsarchive.com.
