# Supplementary material for: Resting parasympathetic activity is associated with malodor‐induced change in perceived foreignness of speakers
Source: Brain Behav. 2023 Sep 21;13(11):e3249. doi: 10.1002/brb3.3249 (PMC10636398; doi:10.1002/brb3.3249)
Supplement: Supplementary file 1 — Supporting Information [file BRB3-13-e3249-s001.pdf]

Supplementary Material

Speaker Stimuli

Audio recordings of speakers reading the story "Comma Gets a Cure" (Copyright 2000 Douglas N. Honorof, Jill McCullough & Barbara Somerville) were obtained with permission from the International Dialects of the English Archive (IDEA) ("International Dialects of the English Archive," www.dialectsarchive.com). The recordings used in this study are listed here by their IDEA ID: Florida 4, Saudi Arabia 4, India 2, Oklahoma 7, Australia 16, Italy 9, Germany 9, Italy 15, Anhui 4, Michigan 13, Scotland 20, Saudi Arabia 5, New York 8, Scotland 19, Russia 6, Czech Republic 3, England 33, Sierra Leone 1, Ohio 7, Chile 5, Spanish 5, Florida 3, India 4, Henan 3, Oklahoma 5, England 34, Australia 14, Russia 12, Spain 6, Jalisco 2, Ukraine 3, Syria 1, South Africa 8, New York 9, Quebec 8, Germany 16.

The 36 audio recordings (18 male, 18 female) were edited to the selection used in Reid et al., (2012): "Even so, on her first morning she felt stressed. She ate a bowl of porridge, checked herself in the mirror and washed her face in a hurry. Then she put on a plain yellow dress and a fleece jacket, picked up her kit, and headed for work." Each clip was edited into 3, 6, or 9 second segments. The start and end points (in milliseconds) of each clip were determined using a random number generator, so that each comprised a slightly different portion of the script. Both male and female speakers were used in an effort to use a balanced stimuli set, and to investigate whether perceptions of speakers are influenced by whether they are of the same or opposite sex of an individual, as perception of vocal attractiveness been implicated in research on mate preferences (Pisanski et al., 2014). The duration of the audio recording was additionally manipulated, as extant research has demonstrated that the duration of an audio stimulus

influences perception (Carcone et al., 2015; Fowler, Lilienfeld, & Patrick, 2009), and provides evidence that individuals can recognize that a speaker has an accent after listening to as few as four words (Hailstone et al., 2012). Therefore, we used of a range of durations to examine how the effect of time exposed to a speaker's voice affects perceptions.

300 workers on Amazon's Mechanical Turk (Mturk) completed ratings of the audio recordings in order to create two sets matched in foreignness, sex, and duration to use in the main study. Each Mturk participant was asked to rate 5 recordings to limit effects of habituation. They were asked "How foreign does this speaker sound? (1=not at all foreign, 9=very foreign)", "How comprehensible is the speaker? (1=very difficult to understand, 9=very easy to understand)", whether the speaker was male or female, and which word of four choices was spoken in the clip. The sex of the speaker and word choice questions were used to evaluate attention to the recording. Of the 300 participants, 188 were male, 112 female; the mean age was 32.5, SD 10.1; 78% were White, 9% Asian, 7% Black, 3% Bi- or Multiracial, 3% Hispanic, 3% American Indian. Participants that incorrectly identified the sex of the speaker or the word spoken in the recording were excluded from the ratings for that recording (n=31). Participants that reported a language other than English as their native language were excluded from all ratings (n=7). Additionally, any workers not originating from the United States were excluded from all ratings (n=1). Each recording was rated by an average of 34 workers (SD 2.2). After the ratings were complete, recordings were split into two sets of 18 speakers that did not differ in perceived foreignness (t_{34}= 0.170, p=.866). This analysis was repeated with the University of Chicago sample reported in the manuscript which confirmed that there was no significant difference in perceived foreignness between speakers sets (t_{34}= .043, p=.966). Further, all reported analyses remained unchanged when controlling for speaker set presentation order.

Assessing Model Fit

Chi-square goodness-of-fit tests using the car package in R were calculated to examine whether adding item-level random intercepts and random slopes improved model fit for the HLM examining the effect of condition (odor/control) and speaker group (ingroup/outgroup) on perceived foreignness. Goodness-of-fit tests were supplemented by power simulation analyses through the simr R package (Green & MacLeod, 2016) (1000 simulations per analysis) to decide on the model to be presented in the manuscript. Results show that adding item-level random intercepts, and random slopes, respectively, to the model did significantly improve model fit for perceived foreignness, however the random slopes model was not sufficiently powered (Table S1). Therefore, the HLM described in the manuscript implements random intercepts at the subject- and item-level, but no random slopes.

Manipulation Check for Baseline Group Differences

As a manipulation check, we conducted an HLM examining the effect of condition (odor/control) and speaker group (ingroup/outgroup) on perceived foreignness at baseline to make sure subsequent results in post-odor manipulation data were not likely to be driven by preexisting differences in the odor and control conditions. Results show no existing baseline differences in foreignness (interaction effect: $\beta = -0.03$, $p = .605$) between ingroup and outgroup speakers as a function of experimental condition (odor/control).

Additional Analyses

Additional HLMs were run to examine whether the principal finding that participants in the odor condition demonstrated higher foreignness ratings of outgroup speakers and lower foreignness ratings of ingroup speakers compared to control participants was moderated by the

sex of the speaker or the duration of the audio recording. A HLM examining the effects of condition (odor/control), speaker group (ingroup/outgroup) and sex of speaker (male/female) on perceived foreignness was not significant (interaction effect: $\beta = 0.09$, $p = .377$, 95% CI [-0.11, 0.28]), showing that the effect of the odor manipulation and speaker group on perceived foreignness was not influenced by the sex of the speaker. A HLM examining the effects of condition (odor/control), speaker group (ingroup/outgroup) and duration of recording (3,6,9 seconds) on perceived foreignness was not significant (interaction effect: $\beta = 0.02$, $p = .276$, 95% CI [-0.02, 0.06]), showing that the effect of the odor manipulation and speaker group on perceived foreignness was not influenced by the length of time exposed to the speakers' voices.

We conducted additional linear models to test whether negative affect or emotional arousal may explain the difference in perceived foreignness of speakers due to the odor manipulation. Participants rated a series of unpleasant pictures from the International Affective Picture System (IAPS) (Lang, Bradley, & Cuthbert, 2008) in perceived negativity and emotional arousal during the odor manipulation, in addition to rating the accented speakers. A linear model examining the effect of condition (odor/control) on perceived negativity of unpleasant pictures was not significant ($\beta = -0.13$, $p = .638$, 95% CI [-0.65, 0.40]), showing that our measure of negative affect was not affected by the odor manipulation. Similarly, the linear model examining the effect of condition (odor/control) on perceived emotional arousal of unpleasant pictures was not significant ($\beta = 0.07$, $p = .781$, 95% CI [-0.46, 0.60]), demonstrating that emotional arousal was not impacted by the odor manipulation. We also examined negativity and emotional arousal ratings as statistical moderators in the HLM testing the effect of condition (odor/control) and speaker group (ingroup/outgroup) on perceived foreignness. A HLM examining the effect of condition (odor/control), speaker group (ingroup/outgroup), and negativity of unpleasant pictures

was not significant (interaction effect: β = -0.04, p = .408, 95% CI [-0.14, 0.06]), showing that our measure of negative affect does not explain results. Similarly, the linear model examining the effect of condition (odor/control), speaker group (ingroup/outgroup), and emotional arousal of unpleasant pictures was not significant (interaction effect: β = -0.06, p = .246, 95% CI [-0.16, 0.04]), demonstrating that emotional arousal does not explain results. These analyses suggest that our findings were not due to negative affect or emotional arousal from the odor.

References

Berntson, G. G., Bigger, J. J., Eckberg, D. L., Grossman, P., Kaufmann, P. G., Malik, M., Nagaraja, H. N., Porges, S. W., Saul, J. P., Stone, P. H., van der Molen, M. W. (1997). Heart rate variability: origins, methods, and interpretive caveats. *Psychophysiology*, 34(6), 623–48.

Cacioppo, J. T., & Berntson, G. G. (1994). Relationship between attitudes and evaluative space: A critical review, with emphasis on the separability of positive and negative substrates. *Psychological Bulletin*, 115(3), 401–423.

Carcone, A. I., Naar, S., Eggly, S., Foster, T., Albrecht, T. L., & Brogan, K. E. (2015). Comparing thin slices of verbal communication behavior of varying number and duration. *Patient Education and Counseling*, *98*(2), 150–155.

Duncan, L. A., Schaller, M., & Park, J. H. (2009). Perceived vulnerability to disease: Development and validation of a 15-item self-report instrument. *Personality and Individual Differences*, *47*(6), 541–546.

Fowler, K. A., Lilienfeld, S. O., & Patrick, C. J. (2009). Detecting psychopathy from thin slices of behavior. *Psychological Assessment*, *21*(1), 68–78.

Green, P., MacLeod, C.J. (2016) simr: an R package for power analysis of generalized linaer mixed models by simulation. Methods in Ecology and Evolution, 7(4), 493-498.

Hailstone, J. C., Ridgway, G. R., Bartlett, J. W., Goll, J. C., Crutch, S. J., & Warren, J. D. (2012). Accent processing in dementia. *Neuropsychologia*, *50*(9), 2233–44.

Lang, P. J., Bradley, M. M., & Cuthbert, B. N. (2008). International affective picture system (IAPS): Affective ratings of pictures and instruction manual. Technical Report A-8.

Makhanova, A., Miller, S. L., & Maner, J. K. (2014). Germs and the out-group: Chronic and

situational disease concerns affect intergroup categorization. *Evolutionary Behavioral Sciences*, *9*(1), 8–19.

Navarrete, C. D., & Fessler, D. M. T. (2006). Disease avoidance and ethnocentrism: the effects of disease vulnerability and disgust sensitivity on intergroup attitudes. *Evolution and Human Behavior*, *27*(4), 270–282.

Norman, G. J., Norris, C. J., Gollan, J., Ito, T. A., Hawkley, L. C., Larsen, J. T., Cacioppo, J. T., Berntson, G. G. (2011). Current Emotion Research in Psychophysiology: The Neurobiology of Evaluative Bivalence. *Emotion Review*, *3*(3), 349–359.

Pisanski, K., Hahn, A. C., Fisher, C. I., DeBruine, L. M., Feinberg, D. R., & Jones, B. C. (2014). Changes in salivary estradiol predict changes in women's preferences for vocal masculinity. *Hormones and Behavior*, *66*(3), 493–497.

Tybur, J. M., Lieberman, D., & Griskevicius, V. (2009). Microbes, mating, and morality: Individual differences in three functional domains of disgust. *Journal of Personality and Social Psychology*, *97*(1), 103–122.

Tables

Table S1.

Goodness-of-fit and power simulation results for HLMs predicting perceived foreignness

Model	df	AIC	BIC	logLik	deviance	chisq	*p*	power
Subject Only Random Intercepts	6	1517.2	1547.0	-752.60	1505.2			58.6%
Subject + Item Random Intercepts	7	1192.4	1227.2	-589.21	1178.4	326.77	< 0.001	80.1%
Subject + Item Random Intercepts & Random Slopes	11	1036.5	1091.1	-507.25	1014.5	163.93	< 0.001	19.1%

Note. Model fit statistics for the effect of condition and speaker group on foreignness. Model analyses were performed iteratively, and each row reflects a comparison between the given model and that listed in the row above.

Table S2.

Means and standard errors for individual difference measures between experimental conditions

	Control		Odor	
	Mean	SEM	Mean	SEM
Age	21.487	0.359	20.961	0.324
Total Disgust	3.558	0.170	3.813	0.142
Pathogen Disgust (subscale)	3.867	0.190	4.224	0.135
Moral Disgust (subscale)	4.044	0.229	4.157	0.188
Sexual Disgust (subscale)	2.763	0.212	3.057	0.231
Perceived Vulnerability to Disease (PVD)	3.552	0.149	3.647	0.128
Perceived Infectability (PVD subscale)	3.473	0.190	3.448	0.162
Germ Aversion (PVD subscale)	3.621	0.198	3.821	0.197
Resting HF-HRV	6.327	0.232	6.272	0.285
HF-HRV Reactivity	6.370	0.144	6.150	0.201

Note. Table S2 lists means and standard errors for the odor and the control conditions. Independent groups t-tests revealed no significant differences between conditions were detected (all *ps* > 0.05).

Table S3.

Means and standard deviations for ingroup and outgroup speaker count by trial and condition

Trial	Condition	Group	Speaker Count Mean	Speaker Count SD
Baseline	Control	Ingroup	7.31	1.67
		Outgroup	10.69	1.67
	Odor	Ingroup	7.60	1.83
		Outgroup	10.40	1.83
Post-Odor	Control	Ingroup	7.14	2.37
		Outgroup	10.86	2.37
	Odor	Ingroup	7.83	2.42
		Outgroup	10.17	2.42

Note. Table S3 lists the means and standard deviations for the number of speakers identified as ingroup and outgroup members for the odor group and the control group during baseline and post-odor manipulation trials.
